# Bleeding After a Single Dose of Ketorolac in a Postoperative Patient

**DOI:** 10.7759/cureus.8919

**Published:** 2020-06-30

**Authors:** Anupam K Gupta, Barbara M Parker

**Affiliations:** 1 Minimally Invasive Surgery, University of Miami Hospital, Miami, USA; 2 Clinical Pharmacy, AdventHealth Orlando and Rockledge Regional Medical Center, Orlando, USA

**Keywords:** gi bleed, ketorolac, general surgery, abdominal bleed

## Abstract

A 74-year-old male patient with extensive cardiac history and hepatitis on apixaban and clopidogrel presented with adhesive small bowel obstruction. The patient underwent an exploratory laparotomy and adhesiolysis for small bowel obstruction. On postoperative day 6, after a single dose of ketorolac, the patient had an intra-abdominal bleed requiring exploratory laparotomy and washout.

## Introduction

There are case reports of patients bleeding postoperatively from ketorolac use; however, meta-analysis has refuted that concern [1,2]. Opioids are efficacious analgesics, but they come with side effects like constipation and drug abuse. There is an increasing tendency to use nonsteroidal medication to avoid the opioid side effect [1,2]. Ketorolac, like other nonsteroidal anti-inflammatory agents (NSAIDs), inhibits the enzyme cyclooxygenase (COX) and does not cause constipation or dependence [1]. In a fraction of patients who were more prone to bleeding on therapeutic anticoagulation with hepatitis, it is not definitive whether ketorolac is safe [1,3-5]. The purpose of this case report is to report a case of postoperative intra-abdominal bleed requiring surgical intervention after a single dose of ketorolac.

## Case presentation

A 74-year-old Caucasian male patient with a weight of 107 kg, and body mass index (BMI) of 33, presented with an adhesive small bowel obstruction. The patient had a history of hypertension, hyperlipidemia, insulin-dependent diabetes mellitus, hypothyroidism, atrial fibrillation, prior deep vein thrombosis, and pulmonary embolism. In addition, there was a history of autoimmune hepatitis, psoriasis, gastroesophageal reflux disease, and obstructive sleep apnea. The patient's past surgical history included multiple cardiac stents, a transcatheter aortic valve replacement, and a thyroidectomy. The patient had no prior history of bleeding disorders. His daily medications included oral antihypertensives, insulin, and levothyroxine, apixaban (5 mg twice daily), and clopidogrel (75 mg daily). The trial of conservative management was not successful, and the patient underwent exploratory celiotomy, adhesiolysis, and small bowel resection for intraoperative enterotomy during adhesiolysis. He had a CHA2DS2-VASc (Congestive heart failure, Hypertension, Age > 65 = 1 point, Diabetes, previous Stroke/transient ischemic attack) score of 7.

**Figure 1:**
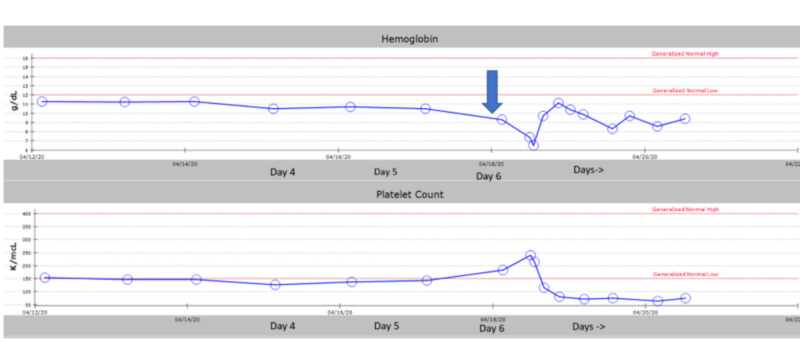
The arrow showing day of administration of ketorolac 15 mg intravenously on postoperative day 6 g/dl, grams per deciliter; K/mcl, thousands per microliter

**Figure 2:**
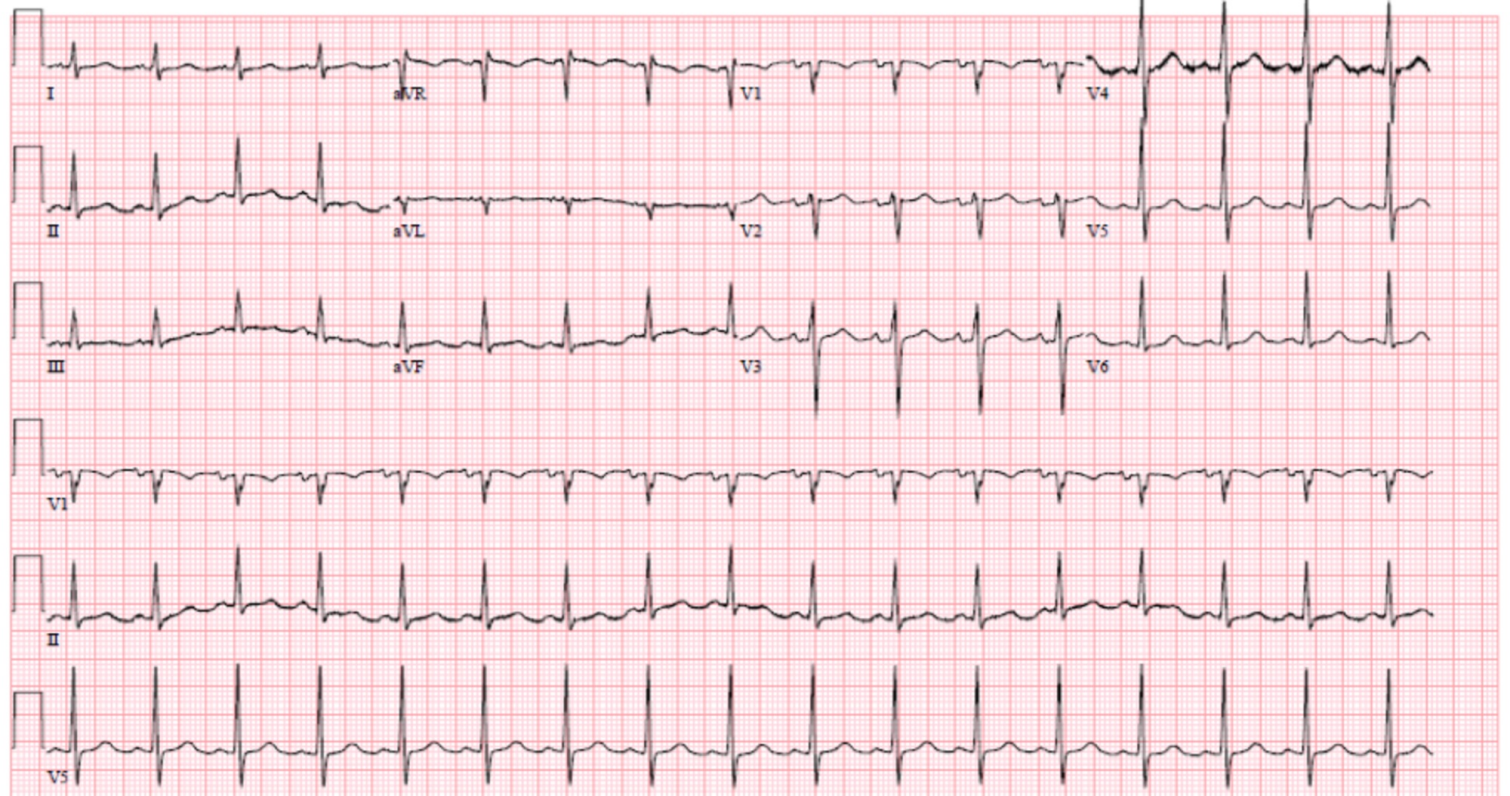
Electrocardiogram showing sinus tachycardia

On postoperative day 1, the patient was anticoagulated with therapeutic enoxaparin. The patient was on carvedilol for blood pressure control and on sliding scale insulin for diabetes among other medications. The patient remained nil by mouth because of an ileus. On the morning of postoperative day 6, the patient's vitals showed a heart rate between 60 and 80 beats per minute (bpm), a blood pressure of 120/60 mmHg, and a saturation of 97% on room air. The patient was given a single dose of 15 mg intravenous ketorolac in the early morning. His initial blood work showed a hemoglobin of 9.3 g/dL with a hematocrit of 28.8% and a platelet count of 183,000/microliter (Figure 1). His serum electrolytes were within normal limits. Three hours after administration of ketorolac, the patient had a drop in blood pressure to 86/60 mmHg from a baseline of 110-130/50-90 mmHg with an associated tachycardia and heart rate of 90-110 bpm (Figure 2).

**Figure 3:**
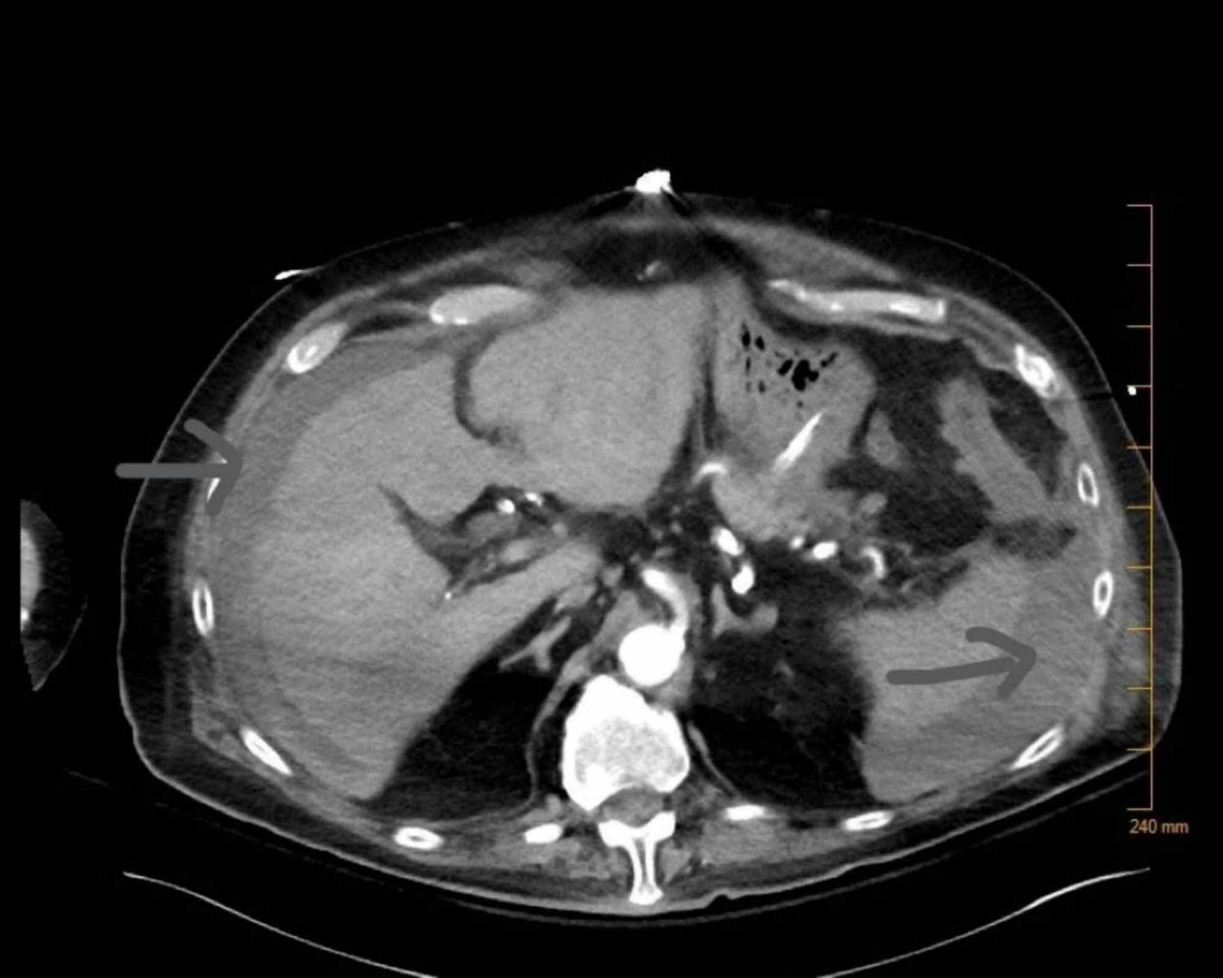
CT showing blood in the abdomen

An electrocardiogram (EKG) performed at the time showed sinus tachycardia. His blood work was significant for a drop in hemoglobin to 7.4 g/dL and hematocrit to 24%. CT of the abdomen and pelvis with intravenous contrast showed fluid collection and evidence of ileus (Figure 3). The patient underwent emergency exploratory laparotomy, which revealed hemoperitoneum with no apparent source of the bleed. The hemoperitoneum was evacuated, and the abdomen was left open with a wound vac (vacuum-assisted closure). The patient was transferred to the intensive care unit (ICU) and resuscitated. The patient continued to have sanguineous discharge from his wound vac, which improved over the next 48 hours after resuscitation with packed red blood cells (RBCs) and platelets. The patient's platelets dropped from a baseline of around 183,000/microliter to 80,000/microliter postoperatively. After adequate resuscitation, the patient underwent a relook laparotomy, and there was no further bleeding. Over the following days, the patient's blood work normalized, and over the next four days, he had the return of his bowel function.

## Discussion

NSAIDs inhibit the COX enzyme, which converts arachidonic acid into prostaglandins and thromboxanes (prostanoids) [6]. Two isoforms of COX exist: COX-1 and COX-2. Ketorolac is an NSAID, which inhibits both COX-1 and COX-2 to varying degrees [1,6]. The COX-1 isoform is present in the gastric mucosa, vascular endothelium, and in platelets. Ketorolac's primary mechanism of action works on the COX-1 pathway, and through COX-1 inhibition, ulceration risk increases, and a reduction in platelet-mediated blood clotting occurs. Ketorolac's analgesic and anti-inflammatory effects originate from the COX-2 isoform [7]. On COX-2 inhibition by ketorolac, the production of prostaglandins reduces pain and inflammation [1,6,7].

Ketorolac's most common side effects include gastrointestinal irritation, nausea, and impairment of platelet aggregation [1,8]. These side effects are dose-dependent, with higher doses corresponding to a worse side effect profile [1]. Older patients and ketorolac length of therapy exceeding five days also increase adverse effect likelihood [1,9].

Ketorolac in clinical settings has a higher analgesic to anti-inflammatory ratio compared to other NSAIDs [1]. Ketorolac reduces opioid use via literature review multistudy analysis by 9%-66% [1,2]. For instance, data suggest that although the onset of ketorolac is slower than that of the opioid morphine, the effectiveness is similar and duration longer [10]. Pharmacokinetically, ketorolac has an intermediate half-life of five to six hours, and timing is essential as its onset may take 30 to 60 minutes with an opioid-sparing effect taking place around four hours after administration [9]. Platelet function does not return to normal until 24 to 30 hours (five half-lives) after a single dose.

Blood clotting is not directly affected by NSAIDs. However, ketorolac inhibits thromboxane A2 and prostacyclin, which are vital to clot formation [9]. A study of animals injected with ketorolac demonstrated a decrease in platelet aggregation (10% vs. 75% for controls), and significant bleeding time (291±5 seconds) [7]. In another study, 40 volunteers on single oral doses from 2.5 to 200 mg showed increased bleeding time and inhibition of platelet aggregation three hours after administration [1]. Twenty-four hours after these doses, bleeding time remained elevated in patients having taken doses higher than 15 mg.

Acute renal failure after ketorolac administration is reversible after discontinuation of the drug. Acute renal failure is a dose-related phenomenon, and renal failure tends to occur more frequently in administration times of five days or more [11-13]. It has primarily been shown that single doses of parenteral ketorolac do not cause an increase in the incidence of adverse cardiovascular, gastrointestinal, or renal adverse outcomes in older adults [14]. However, in our case, this patient bled significantly after a single conservative dose of 15 mg of ketorolac. NSAIDs, in addition to anticoagulation, as enoxaparin in this case, increase the risk of bleeding. Our elderly patient was susceptible and sensitive to the addition of ketorolac as three hours after ketorolac 15 mg intravenously once, his platelets dropped more than 50% from his baseline (183 to 80 k/mL). Subsequently, his hemoglobin dropped from 9.3 to 7.4 g/dL (Figure 2). An additive effect of enoxaparin accumulation due to a significant renal function decline may also have exacerbated his bleeding. It is unlikely, in this case, that ketorolac caused acute kidney injury as this usually ensues after several doses of the drug, not just one, as a possible contributor. Renal blood flow is dependent in part on prostaglandins, especially during periods of stress when the renin-angiotensin system is activated [11,12]. It is plausible that ketorolac may contribute to renal failure in elderly patients, those with heart failure, and those exposed to other nephrotoxic agents. Although documented in case reports, clinical studies refute this claim, but these studies are unreliable as they have been few in number and inadequate [15,16].

Ketorolac does not cause hepatic toxicity in surgical patients [3-5]. However, it can cause elevated liver enzymes in up to 1% of individuals, typically an asymptomatic effect. The mechanism behind the ketorolac hepatotoxicity is unknown. It is unlikely that ketorolac itself caused an elevation in liver enzymes in this case, but given this patient's history of autoimmune hepatitis, this fact could have played a role in making him more vulnerable to bleed. An inflamed liver from hepatitis impairs the body's natural clotting cascade pathway through the reduction of coagulation protein synthesis, causing longer clotting times [5].

## Conclusions

Age, renal and hepatic function, concomitant blood thinners, drug interactions, and patient medical history all play a role in patient outcomes postoperatively. Although ketorolac clinically is the preferred choice over opioids after surgery, it is not without its risks in certain patients. Ketorolac, owing to its antiplatelet function in the background of blood thinners and several medical comorbidities, can precipitate bleeding postoperatively.
